# Altruistic motivation or instrumental motivation: The spatial spillover effect of corporate social responsibility on corporate green innovation

**DOI:** 10.1371/journal.pone.0290125

**Published:** 2023-10-19

**Authors:** Doudou Chen, Zhen Peng

**Affiliations:** Hubei University, School of Business, Wuhan, China; University of Almeria: Universidad de Almeria, SPAIN

## Abstract

In recent years, the concept of corporate social responsibility has gained more attention from investors, and green innovation has become a key factor in China’s economic growth. Despite this, regional disparities still remain, and the impact of corporate social responsibility on green innovation in local and surrounding areas is worth exploring. This article uses a Spatial Durbin Model to analyze the spatial spillover effect and mechanism of corporate social responsibility on green innovation of A-share listed companies in China from 2010 to 2020. The results show that corporate social responsibility behavior motivated by "tools" has a negative effect on local enterprises’ green innovation, while also having a negative spillover effect on surrounding areas, thus affecting the spatial pattern of green innovation. Further research suggests that fulfilling corporate social responsibility can attract investors, alleviate financing constraints, and change corporate resource investment strategies, thus promoting cross-regional resource flow and enhancing green innovation. The results of this study remain consistent when the matrix and variables are changed. This article provides new insights into corporate social responsibility and green innovation, and offers policy recommendations to improve green innovation in enterprises.

## 1. Introduction

In order to advance the market-oriented green technology innovation system, speed up the research, production and utilization of advanced energy-saving and carbon reduction technologies, the National Development and Reform Commission and the Ministry of Science and Technology of China, in conjunction with other relevant departments, formulated the "Implementation Plan for Further Improving the market-oriented green technology innovation system (2023–2025)" in 2022. This illustrates that innovation is the primary impetus for industrial progress, and green is the overall form of industrial evolution. Pushing green innovation and development of businesses is an unavoidable trend for future economic growth, and it is also a necessary course for China’s economy to transfer to high-quality growth. Since the reform and opening up, highly polluting industries such as coal-fired power generation and chemical manufacturing have had a major impact on advancing China’s economic advancement, but their expansive development model has also generated numerous environmental pollution issues. To meet the requirements of low-carbon development and achieve the goals of “dual carbon” and “harmonious coexistence between humans and nature”, it is essential to promote technological progress in key industries through technological innovation.

In addition to environmental regulations [1], market competition, and market demand [2], corporate social responsibility is increasingly being seen as a factor in promoting green innovation in enterprises. Studies have shown that by fulfilling their social responsibilities, companies can better meet the needs of their internal and external stakeholders, leading to the development of a wider and deeper network of relationships between the enterprise and its stakeholders [3], and providing beneficial technology and information for internal innovation. However, some argue that fulfilling corporate social responsibility can lead to unnecessary costs and may take away resources from other areas, such as innovation, which could have a negative effect [4]. The debate surrounding corporate social responsibility may be due to the differing motivations behind it. Traditional research on social responsibility suggests that companies are driven by two distinct motivations: altruistic and instrumental [5]. When a company is altruistically motivated, taking on social responsibility is seen as a strategic investment with marketing and reputation benefits, which can lead to increased innovative behavior and green innovation. On the other hand, when social responsibility is used as a tool of flattery, it is often accompanied by “short-sighted” behavior. This is because research and development expenses can reduce current accounting profits, which is not beneficial to the compensation of managers. Thus, management, out of “self-interest” motivation, supports the fulfillment of social responsibility and suppresses innovation to gain short-term benefits. Therefore, it is important to understand the motivations behind corporate social responsibility in order to better understand the relationship between the two.

Different motivations result in different outcomes when it comes to corporate social responsibility, which in turn shapes the logic of the interaction between the two. When a company pursues corporate social responsibility out of a “tool” motive, it is driven by personal desires rather than a genuine concern for the public [6]. This type of behavior is often passive and serves a purpose, such as competing for social resources, including political resources and financing channels. Research has shown that fulfilling corporate social responsibility can send positive signals to the capital market, reduce bond credit spreads, and influence rating agencies’ credit ratings of companies, thus reducing corporate financing costs [7]. It has been overlooked that corporate social responsibility driven by “tool” motivation can have a heterogeneous effect on innovation. This includes taking on environmental and social responsibilities for targeted poverty alleviation, charitable donations, environmental governance, and protecting the local environment, which is in line with the local government’s performance goals and can help companies gain political capital such as government financial subsidies and tax incentives [8]. On the one hand, political connections can stimulate corporate technological innovation through government preferential policies [9], but on the other hand, they can also hinder corporate technological innovation due to the “curse effect” of political resources [10]. So, what are the consequences of corporate social responsibility based on self-interest motivation on innovation? Does it accumulate more resources for innovation from a long-term development perspective or suppress the driving force of innovation for short-term benefits? How can enterprises address these difficulties and promote green innovation?

The sources of corporate financing are not restricted to local areas, and the flow of funds across regions is also a major channel for corporate financing. The competition for social resources among companies driven by selfish motives will intensify the spatial flow and allocation of resources, particularly in the political connection between government and businesses, which may further increase the spatial competition of resources by companies and attract more quality resources to the local area, thus forcing some unfavorable economic activities to move to nearby areas, further affecting the spatial allocation of innovative elements. With the deepening of reform and opening up, market segmentation and administrative barriers are being broken down, and market links between provinces and regions are becoming closer. The cross-regional flow of factors is becoming more common. Therefore, it is necessary to consider whether the government-enterprise related activities in the process of fulfilling corporate social responsibility will exacerbate the spatial competition of resources, and the resulting spillover effect and spatial imbalance of social responsibility on corporate innovation. The current unbalanced and inadequate regional development in China is still a long-term issue, and spatial location remains an important consideration for businesses. Therefore, this paper will explore if there is a spatial spillover effect in corporate social responsibility and the spatial correlation of corporate innovation behavior, and analyze the impact of corporate innovation behavior on the spatial allocation of innovation factors. Through this study, not only can the effectiveness of corporate innovation driven policies be improved, but it can also provide useful references for the integration and development of enterprises in the region.

This article empirically investigates how corporate social responsibility activities influence the green innovation of local and neighboring businesses. The findings show that when corporate social responsibility is implemented through “tools”, it has a detrimental effect on the local enterprises’ green innovation, while creating negative externalities in the surrounding areas, which in turn alters the spatial pattern of green innovation. Subsequent studies have identified three pathways through which corporate social responsibility affects the spatial effects of green innovation: reinforcing ties between business and government, reducing financing difficulties, and enhancing external regulatory efficiency.

In comparison to existing literature, this article stands out in the following aspects: First, existing research on corporate social responsibility and innovation is restricted to local effects, yet inter-regional factor flows and policy spillovers might bring about spatial associations in corporate social responsibility practices. Thus, this article examines the spatial spillover effect of corporate social responsibility on innovation behavior from a spatial viewpoint. Second, it innovatively explores the motivations for companies to fulfill their social responsibility and how it influences green innovation in companies, as well as the transmission process between the two. This article investigates the influence of corporate social responsibility on innovation via political connections, financing constraints, and external governance factors, using a spatial econometric model. Additionally, it looks into the agglomeration sources and diffusion paths of innovation resources and extends existing studies in order to tackle the issue of conflicting research findings. Third, the article introduces a multi-level spatial weight matrix built using the urban adjacency weight matrix and the block weight matrix corresponding to individual companies, which is used to examine the impact of enterprise and urban heterogeneity on enterprise innovation performance.

## 2. Literature review and hypothesis development

Endogenous growth theory suggests that innovation is driven by investments in capital and talent, and that investment strategies are determined by the collective decisions of shareholders based on their interests. Businesses typically prioritize profits when deciding on their investment strategies, which can affect innovation through differences in resource allocation. Government industrial policies can also influence the spatial distribution of resources and the performance of enterprise innovation. Enterprises may fulfill their social responsibility out of their own self-interest [11], or to comply with external policies and regulations in order to gain political resources. When external factors drive a company to fulfill its social responsibility, based on competitive and legitimacy pressure [12], it will be more motivated to imitate the social responsibility behavior of other companies, leading to a competition for resources and to a spatial correlation in the innovation behavior of the company. To successfully allocate resources, cooperation with government policies is usually necessary. By obtaining and investing advantageous resources, unfavorable resources are transferred to the surrounding areas, which affects the spatial pattern of enterprise innovation. Fulfilling corporate social responsibility can not only build a good corporate image in front of the government and gain politically connected resources, but also attract investors’ attention, expand financing channels, and reduce financing constraints, as well as leverage the external governance effect of institutional investors driven by long-term interests, thus influencing corporate innovation. This article mainly examines the spatial mechanism of corporate social responsibility on innovation effects through three approaches: strengthening government-enterprise connections, alleviating financing constraints, and enhancing external governance effects.

### 2.1. The mechanism of financing constraint

Innovation activities often involve high risks and high switching costs, and the output of such activities is usually intangible assets such as patents and intellectual property rights. Moreover, R&D investments have their own unique characteristics such as requiring a high level of expertise and secrecy, making them more susceptible to information asymmetry and principal-agent problems. The standard order of corporate financing is internal financing, debt financing, and equity financing. However, these internal sources of funds are limited and may not be enough to support the long-term and large-scale investments in innovation activities. Therefore, external financing is often needed, but creditors, such as banks, tend to demand collateral in the form of tangible assets and a steady cash flow for repayment of principal and interest. This creates a tension between the financial requirements and the need for continuous investment in innovation. Consequently, innovative R&D projects often encounter serious financing constraints, leading to insufficient R&D investment. Studies have shown that providing more financial support has a positive effect on enterprise innovation activities.

The disclosure and implementation of corporate social responsibility (CSR) is a crucial way for external investors to access corporate information. It helps to reduce information asymmetry, generate high-quality CSR reports, and enable creditors such as banks and capital market investors to make more accurate predictions about the future performance, risks, and other potential outcomes of listed companies, thereby facilitating their access to external financing. Additionally, it can also attract ethical investors who prioritize CSR over returns. This preference, if reported in the media and by analysts, can draw more attention to the company and reduce financing constraints [13]. With the growth of regional economic integration, the economic agglomeration effect of the region is increasingly significant, leading to a greater competition for capital among local enterprises. Moreover, the improvement of infrastructure like transportation and communication has allowed investors to go beyond local enterprises in their search for the best. This has resulted in an influx of capital to local areas, while also reducing financing opportunities for enterprises in surrounding areas, thereby affecting the spatial pattern of green innovation for businesses. Consequently, this article proposes the first hypothesis:

**Hypothesis 1:** Enterprises can promote financial capital in their local area by meeting their social responsibilities, reducing the financial capital of nearby cities, and facilitating the financing of green initiatives.

### 2.2. The mechanism of external governance

Institutional investors, as external stakeholders of an enterprise, have the incentive to supervise the management and, if necessary, influence the behavior of the enterprise due to their large shareholding. This is because innovation investment is risky and intertemporal, so enterprise managers may be reluctant to make such investments. Institutional investors, however, have a strong economic motivation to get involved in corporate governance activities in order to protect their own interests and reduce investment risks, as well as information asymmetry and moral hazard problems [14]. Additionally, owning numerous company stocks gives them economies of scale when evaluating investments, allowing them to gain a better understanding of the market than individual investors [15]. This means that they have the motivation to evaluate potential long-term returns, rather than short-term fluctuations in prices. As a result, organizations seeking long-term returns will encourage enterprises to invest in innovation.

Institutional investors are likely to make long-term investments in companies that take corporate social responsibility seriously due to the potential liquidity losses from their large and concentrated shareholding [16], as well as the reputation insurance effect of good corporate social responsibility, which helps to reduce risks. Therefore, as a way to reduce potential risks, institutional investors must pay attention to corporate social responsibility when investing in shares. Attention is a limited resource, so investors will only focus on information that captures their attention. However, since their attention is limited, if they focus on one company, it will reduce their attention to other companies in the same area, thus affecting the spatial flow and redistribution of attention resources. This, in turn, will lead to a spatial spillover effect of corporate social responsibility on green innovation in enterprises. As a result, this article proposes the second hypothesis.

**Hypothesis 2:** Companies that embrace corporate social responsibility will draw more institutional investors to themselves, consequently lessening the attention to other enterprises, while encouraging green innovation.

### 2.3. The mechanism of government-enterprise connections

The curse effect of political resources implies that political connections can be detrimental to the independent innovation of businesses [17]. Firstly, forming and sustaining political relations requires a certain expense, comprising of the time cost of entrepreneurs, related transaction costs, and bribes to the authorities (officials); all of which can supplant the resources allocated to enterprise innovation and produce negative effects. Secondly, although firms can gain certain information and policy advantages through political connections, it can weaken the independence of the enterprise’s decision-making, making it simpler for executives to follow politicians. When government requirements are at odds with the business’s development strategies, companies have to forgo some of their autonomy to abide by the government requests and maintain their political ties, which prevents them from making the best decisions that are beneficial for their long-term growth. Moreover, political connections can provide firms with government purchase orders and government projects, which aids them in surviving and gaining convenience in a highly competitive market. However, it can also lead to complacency and reliance on the management team, stifling their creative thinking and long-term investments in innovation, and weakening their enthusiasm to improve performance through innovation [18].

When a company fulfills its social responsibility out of a “tool” motivation, it is likely to comply with external policy orientations for utilitarianism. When there are clear policies, laws, and regulations that require the company to assume social responsibility (such as energy conservation and emission reduction, charitable donations, protecting employee rights, etc.), the company will actively attach social responsibility attributes to prove the legitimacy of its behavior and establish a citizen image of compliance with laws and regulations. This can leave a good impression on government officials and establish political connections. As a result, companies that invest more in social responsibility projects are more likely to gain political convenience. This can also encourage surrounding enterprises to imitate and follow, thereby exacerbating their competition for political resources. With the development of regional integration, administrative barriers are gradually broken, and the flow of political resources is more smooth. This may trigger a spatial spillover effect of corporate social responsibility on green innovation through government enterprise connections. Therefore, it is hypothesized that corporate social responsibility can have a positive effect on green innovation through government enterprise connections.

**Hypothesis 3:** Companies can impede green innovation by meeting their social responsibilities to improve their own and neighboring enterprises’ political connections.

In short, corporate social responsibility can have both a beneficial and a detrimental effect on green innovation. In China, the government’s intervention in the market and its influence on enterprise decisions can affect resource allocation efficiency, and external institutional investors are unable to play their intended role. Furthermore, government subsidies and other advantages can lead to a lack of motivation for enterprises to improve their performance through innovation, thus weakening the incentive effect of market competition on innovation. In conclusion, when the government is too closely connected and does not allow enough independence to the market and enterprises, its inhibitory effect on innovation can outweigh the positive effects of financing constraints and external governance, ultimately having a negative impact on green innovation of enterprises.

## 3. Empirical design

### 3.1. Definition of the main variables is necessary

#### 3.1.1. Corporate social responsibility

This article uses the Hexun Network’s Corporate Social Responsibility Report’s overall score to evaluate the level of commitment to corporate social responsibility. This rating is based on the financial and social responsibility reports of listed companies in China. It is analyzed from five perspectives: shareholder responsibility, employee responsibility, supplier responsibility, customer and consumer rights and interests, environmental responsibility, and public responsibility. Thirteen secondary indicators and thirty-seven tertiary indicators are set up to systematically evaluate businesses’ social responsibility commitment, which can provide a relatively comprehensive view of the fulfillment of corporate social responsibility.

#### 3.1.2. Corporate green innovation

This article evaluates green innovation in businesses by calculating the number of green patent applications (green). To eliminate the issue of the right-biased distribution of green patent data, the sum of green invention patent applications and green utility model applications is added to one, then the natural logarithm is taken. Additionally, the number of green patents acquired by enterprises (green_r) is used as an alternative variable to reinforce the results of the regression.

#### 3.1.3. Earnings quality

This article evaluates the quality of corporate earnings by using accrual earnings management. Generally, earnings management undermines earnings quality. To calculate the absolute value of controllable accrued profits, the Jones model with cross-sectional correction is employed as a substitute variable for earnings quality. Consequently, the higher the level of accrued earnings management in a company, the lower the quality of the company’s earnings. The calculation process is as follows:

First of all, figure out the total accrued profit (TA):

$${TA}_{i,t}={NF}_{i,t}-{OCF}_{i,t}$$

In the formula, Ta_i,t_ stands for the total accrued profit of the company i in year t, NF_i,t_ denotes the operating profit of the same company in the same year, and OCF_i,t_ symbolizes the net cash flow from operating activities of the firm in year t.

Then, divide the sample data into different years and industries, and use regression to obtain parameter values ∝_1_、∝_2_、∝_3_ for each year and industry.


$$\frac{{TA}_{i,t}}{A_{i,t-1}}=\propto_{1}\frac{1}{A_{i,t-1}}+\propto_{2}\frac{\Delta {REV}_{i,t}}{A_{i,t-1}}+\propto_{3}\frac{{PPE}_{i,t}}{A_{i,t-1}}+\varepsilon_{i,t}$$

In the formula, *A*_*i*,*t*−1_ represents the total assets of the company i in the previous year, t-1, while B indicates the change in the main business income of the company i between year t and t-1. Furthermore, *PPE*_*i*,*t*_ stands for the original book value of the fixed assets of the company i in year t. The model also incorporates a constant term to the traditional estimation equation, which helps reduce heteroscedasticity and counterbalance the measurement bias caused by the lack of scale variables in the model. Furthermore, the parameter values obtained through regression are denoted by ∝_1_、∝_2_、∝_3_.

To determine the Non Discretionary Accruals (NDA), the parameter values for each year and industry are inserted into the formula:

$${NDA}_{i,t}=\propto_{1}\frac{1}{A_{i,t-1}}+\propto_{2}\frac{\Delta {REV}_{i,t}-\Delta {REC}_{i,t}}{A_{i,t-1}}+\propto_{3}\frac{{PPE}_{i,t}}{A_{i,t-1}}$$

Δ*REC*_*i*,*t*_ symbolizes the transformation in accounts receivable of the company in year t and year t-1, with all other interpretations staying the same.

Last, the Non Discretionary Accruals (NDA) is reduced by the Total Accruals (TA) to get the Discretionary Accruals (DA).


$${DA}_{i,t}=\frac{{TA}_{i,t}}{A_{i,t}}-{NDA}_{i,t}$$

Among them, NDA_i,t_ stands for the Non Discretionary Accruals of company i in year t, while DA_i,t_ is the Discretionary Accruals of the same company in the same year, and is taken as an absolute value to represent earnings quality (AEM).

#### 3.1.4. Financing constraint

Following the approach of [19], a comprehensive measurement index, the KZ index, is created as a proxy variable for financing constraints. Nevertheless, [20] discovered that the dependent and independent variables of the KZ index construction process make use of certain quantitative information of the enterprise, leading to model estimation bias. Consequently, this paper also opts WW index, which is constructed by GMM estimation of investment Euler equation [21], as a substitute variable to improve the robustness of the estimation results.

#### 3.1.5. external governance effects

This article uses the shareholding ratio of institutional investors(INST) and the number of independent directors (Indep) as proxies for external governance effects.

#### 3.1.6. Political connection

This article uses the executive information column from a company’s financial report to determine if the company has any political connections. A dummy variable, Corporate Political Affiliation, is used to measure if the executives of the listed company are former or current government officials, representatives of the National People’s Congress, or members of the Chinese People’s Political Consultative Conference. If so, the value is set to 1, otherwise 0. Additionally, a Political Affiliation Level (PCLevel) is created as an alternative variable, which is an ordered variable. This variable is based on the administrative level of the government agency that is connected to the executives, and is set to 4, 3, 2, or 1 depending on four categories: ministerial, office, department and section level.

#### 3.1.7. Control Variables

In order to control and minimize the influence of other factors on the effectiveness of green innovation in enterprises, this paper has selected ROA(Return of Assets), Lev(Financial leverage), the growth rate of main business income(Growth), operating cash flow(Cashflow), Enterprise size(Size),the proportion of shares held by the largest shareholder (Top1), duality of COB and CEO (dual), independence of the board of directors(Indep), and TobinQ as the control variables.

### 3.2. Spatial effect recognition strategy

By utilizing a spatial econometric model, this paper seeks to identify the correlation between corporate social responsibility and innovation, taking into account both local effects and spatial heterogeneity. However, the macro geographic weight matrix based on traditional urban longitude and latitude is unable to detect the spatial correlation of micro individuals. Consequently, the article applies both OLS and SDM based on special parameters to estimate the relationship between corporate social responsibility and green innovation behavior.

The spatial multi-level model is represented by Eqs (1) and (2).


$$y_{pit}=\alpha +\rho \sum_{j=1}^{n}{w_{pij}y}_{pjt}+{\beta x}_{pit}+\theta \sum_{j=1}^{n}w_{pij}x_{pit}+\beta {*x}_{it}+\mu_{i}+\gamma_{t}+u_{pit}$$ (1)

$$u_{pit}=\lambda \sum_{j=1}^{n}{w_{pij}u}_{pjt}+\varepsilon_{pit}$$ (2)

Among them, *y*_*pit*_ is the innovation level of enterprise p in city t in year i, *x*_*pit*_ is the core explanatory factor of this piece, and *x*_*it*_ is the covariate vector at the enterprise level. The block adjacency weight matrix *w*_*pij*_ is derived from *w*_*ij*_, while *ρ* and *θ* are two spatial lag parameters that quantify the spatial dependence of the dependent variable y and the independent variable x respectively. Moreover, *λ* is the spatial Error term autocorrelation parameter, which captures the spatial dependence of unobserved elements, and *μ*_*i*_ and *γ*_*t*_ are the urban and year fixed effect sets respectively. Lastly, robust standard error is utilized and *u*_*pit*_ is the random Error term.

If *ρ*, *θ* and *λ* are all 0, Eq (1) is based on the local direct effects estimated by OLS. When *λ* = 0, Eq (1) indicates the spatial effect of the SDM model. When *θ* and *λ* are 0, Eq (1) displays the spatial autocorrelation of SLM estimation of enterprise innovation level. Additionally, when *ρ* and *θ* are both equal in Eq (1), Eqs (1) and (2) demonstrate the spatial correlation of SEM model estimation of unobserved factors.

### 3.3. Building a multi-layered weight matrix

This research combines the macro Prefecture-level city matrix and the micro individual matrix to generate a block weight matrix to examine the influence of corporate social responsibility on corporate green innovation from a spatial viewpoint. Building on Dong and Harris’ (2015) work, this paper uses a block Adjacency matrix *w*_*pij*_ to account for individual diversity. The block weight matrix *w*_*pij*_ is constructed based on the extended Prefecture-level city weight matrix *w*_*ij*_, and the size of each block is determined by the number of listed enterprises in the city obtained by the analysis sample in this paper. Consequently, the matrix *w*_*pij*_ is able to capture the spillover effects of individual heterogeneity at different spatial levels [22, 23].

This study uses micro enterprise data to analyze the impact of corporate social responsibility behavior on corporate green innovation. Therefore, a model needs to be constructed to identify the spatial correlation of corporate innovation behavior. To do this, a balanced Panel data will be obtained from listed companies and the sample size of each city will be calculated to create a matching base. Then, the Prefecture-level city matrix will be expanded according to the matching base, with each row and column containing the same values as the city with 100 observations. This will generate a block weight matrix based on the Prefecture-level city weight matrix, with each block size corresponding to the number of listed enterprises in the Panel data being analyzed.

To analyze the impact of corporate social responsibility behavior on corporate green innovation, this study utilizes micro enterprise data to construct a matrix *w*_*pij*_ that links individual dimensions to Prefecture-level cities, thereby identifying the spatial correlation of corporate innovation behavior. From the data of listed companies, a balanced Panel data is first obtained to be analyzed, and the sample size of each city is then calculated to obtain a matching base. Then the matrix of the Prefecture-level city is extended according to the matching base,which is *w*_*ij*_. For example, if there are 100 observed values in city i, the values in row i and column j of the matrix *w*_*ij*_ will be copied and pasted 99 times, creating the block weight matrix *w*_*pij*_ based on the weight matrix of Prefecture-level city. *p* is the listed enterprises of city i, and the size of each block is equal to the number of listed enterprises of the Panel data to be analysed in that city i.

The block weight matrix presents a challenge in obtaining complete information for each city, as not all cities have listed companies. If most Prefecture-level cities are not included in the analysis data and weight matrix, the spatial effect of these cities will be incomplete, as they exist in isolation without any listed enterprises, thus not being able to capture the spatial effect of neighboring cities. This article creates a virtual enterprise p without individual information in each unsampled city, and then calculates the average feature value of listed companies in that province. This average value is used to fill in the virtual enterprise data of unsampled cities. Since the adjusted observation samples are nested in all Prefecture-level cities, the corresponding block weight matrix *w*_*pij*_ is adjusted to reflect the repeated geographical information characteristics of one row and one column for non-sampled cities and multiple rows and columns for sampled cities, based on the distribution of listed enterprises in cities and the original weight matrix of 286 Prefecture-level cities. The weight matrix *w*_*pij*_ obtained in this paper, along with the adjusted analysis sample, contains the complete information of all Prefecture-level cities, thus ensuring that each city has at least one observation value and that the corresponding enterprise level block weight matrix *w*_*pij*_ can accurately capture the complete spatial effect.

### 3.4. Analysis and discussion of empirical results

#### 3.4.1. Descriptive analysis of variables

Table 1 reveals the descriptive statistical results of the main variables. The average number of green innovation applications from enterprises is 14.70 with a standard deviation of 71.40, while the average number of green patents obtained is 2.713 with a standard deviation of 14.09. This implies that there are considerable disparities in innovation inputs and outputs among the enterprises in the sample. Additionally, the mean corporate social responsibility score (CSR) is 14.70 with a standard deviation of 71.40, indicating that the overall social responsibility performance of the companies in the sample is satisfactory but still varied. The parameter distribution of other control variables also demonstrates discrepancies between banks.

**Table 1:** Descriptive statistics of variables.

Variables	Sample	Mean	Median	Standard deviation	min	range
Green	8000	14.70	1	71.40	0	1577
Green_r	8000	2.713	0	14.09	0	499
CSR	8000	27.50	23.02	18.05	-11.29	101.5
DCA	8000	0.0150	0.0140	0.146	-4.343	7.707
KZ	8000	1.843	1.963	1.827	-8.254	18.10
WW	8000	-1.121	-1.051	5.826	-522.0	521.3
INST	8000	0.474	0.489	0.220	0	1.515
Indep	8000	0.374	0.357	0.0570	0.167	0.633
PC	8000	0.358	0	0.480	0	1
PClevel	8000	1.135	0	1.611	0	4
Size	8000	23.00	22.87	1.388	19.08	9.559
ROA	8000	0.510	0.520	0.186	0.00700	1.949
ROE	8000	0.0380	0.0320	0.0540	-0.894	1.570
Cashflow	8000	0.0460	0.0450	0.0700	-0.556	1.217
Growth	8000	2.101	0.0980	166.4	-0.875	14884
TobinQ	8000	1.675	1.364	0.986	0.674	16.19
Dual	8000	0.179	0	0.383	0	1
Top1	8000	0.360	0.343	0.155	0.0360	0.827

#### 3.4.2. Assessing the motivation for CSR

Table 2 reveals that there is a significant positive correlation between corporate social responsibility and overall earnings management level at the 1% level, the coefficient is 3.803. Additionally, a significant positive correlation is observed between corporate social responsibility and positive earnings management level at the 5% level, the coefficient is 1.132. while a significant negative correlation is found between corporate social responsibility and negative earnings management level at the 5% level, the value of coefficient is -7.206. This is measured by the absolute value (AEM), and the parts greater than 0 and less than 0 (as shown in columns (1), (2), and (3)) of the discretionary accruals (DA). To eliminate the COVID-19’s impact on the correlation between corporate social responsibility and earnings management, the observed values in 2019 and 2020 in the research sample were deleted. When the model was regressed again, a positive relationship between corporate social responsibility and earnings management was still observed in column (4) and the coefficient has a value of 3.182. It can be concluded that hypothesis 1 was supported, suggesting that companies that engage more in corporate social responsibility have a greater degree of earnings management. This implies that corporate social responsibility is a form of opportunistic behavior that masks and covers up improper earnings manipulation and other activities that benefit management. This indicates that the implementation of corporate social responsibility is not driven by value appreciation, but rather by the personal interests of management. Management may be more inclined to fulfill social responsibility requirements due to external policy environments. Innovation activities and corporate social responsibility are part of a business’s competitive strategic decisions, and the high risk and unpredictability of innovation can lead to short-term thinking among management, who may be unwilling to invest in innovation. Consequently, this article will further investigate whether corporate social responsibility motivated by self-interest can crowd out innovation activities.

**Table 2:** The influence of CSR on earnings management.

	(1)	(2)	(3)	(4)
	AEM	DA>0	DA<0	AEM
CSR	3.803***	1.132**	-7.206**	3.182**
	(1.018)	(0.564)	(2.553)	(1.104)
Size	-0.007*	-0.007*	-0.003	0.002
	(0.004)	(0.004)	(0.010)	(0.006)
Lev	0.037**	0.032**	0.007	0.038*
	(0.016)	(0.013)	(0.030)	(0.019)
Roa	0.001	1.004***	0.576***	0.149**
	(0.046)	(0.059)	(0.045)	(0.062)
Cashflow	-0.233***	-1.009***	-0.492***	-0.267***
	(0.037)	(0.057)	(0.052)	(0.045)
Growth	0.000***	0.001*	-0.000***	0.000***
	(0.000)	(0.001)	(0.000)	(0.000)
TobinQ	0.004***	-0.000	-0.006**	0.003**
	(0.001)	(0.001)	(0.002)	(0.001)
Dual	0.005	0.001	-0.011	0.006
	(0.005)	(0.005)	(0.009)	(0.007)
Top1	0.012	0.018	-0.014	0.025
	(0.023)	(0.021)	(0.048)	(0.029)
Fixed Year	√	√	√	√
Fixed City	√	√	√	√
N	13387	7973	5414	10679
R^2^	0.018	0.383	0.043	0.017

Note: The robust standard errors are used in the estimation.

* represents significance at the 10% significance level

** represents significance at the 5% significance level

*** represents significance at the 1% significance level.

#### 3.4.3. Direct effect

Table 3 presents the results of Fixed Effect Model of Panel data without incorporating the effect of spatial spillover. The first column displays the results of the core variables, the regression coefficient is equal to -103.943. While the second column adds a number of control variables to reduce any distortions in the causal effects, getting the regression coefficient -54.783. In column (3), ESG is used as a substitute for CSR, while column (4) tests the sum of green inventions and green utility models acquired in the same year (green_r) instead of the sum of green inventions and green utility models applied in the same year (green) to enhance the reliability of the results. All the results are significantly negative at the 1% level. The possible reason for this is that in order to meet customer expectations and related interests through CSR, enterprises need to invest a lot of resources, which can lead to them becoming overly reliant on existing customers and locking themselves into existing customer relationships. This can reduce their ability to sense and understand the needs of potential customers or new trends in customer demand, thus weakening their ability to identify market technological innovation opportunities and reducing their technological innovation performance. This has a “crowding out effect” on green innovation, and with the development of integration, market segmentation and administrative barriers, the weakening effect of corporate social responsibility on green innovation has a spatial effect. Therefore, this article will further examine the relationship between the two from a spatial perspective.

**Table 3:** The impact of CSR on green innovation in local enterprises.

	(1)	(2)	(3)	(4)
	green	green	green	green_r
CSR	-103.943***	-54.783***		-36.156***
	(6.475)	(6.513)		(4.484)
ESG			-0.005***	
			(0.001)	
Size		0.609***	0.575***	0.208***
		(0.031)	(0.031)	(0.017)
Lev		-0.493***	-0.413***	-0.148**
		(0.111)	(0.112)	(0.067)
ROA		-0.453**	-0.467**	-0.162*
		(0.155)	(0.156)	(0.096)
Cashflow		0.173*	0.143	0.038
		(0.104)	(0.104)	(0.066)
Growth		-0.010	-0.010	-0.003
		(0.007)	(0.007)	(0.002)
TobinQ		-0.006	-0.005	-0.005*
		(0.006)	(0.006)	(0.003)
Dual		-0.035	-0.045	-0.029
		(0.029)	(0.029)	(0.018)
Top1		-0.452**	-0.404**	-0.382***
		(0.176)	(0.175)	(0.095)
Fixed Year	√	√	√	√
Fixed City	√	√	√	√
N	13905	13905	12411	13905
R2	0.037	0.184	0.165	0.076

Note: The robust standard errors are used in the estimation.

* represents significance at the 10% significance level

** represents significance at the 5% significance level

*** represents significance at the 1% significance level.

#### 3.4.4. Spatial effect

Table 4 presents the results of the spatial Durbin model’s estimation of the effect of corporate social responsibility on local green innovation. The estimated coefficient of corporate social responsibility was found to be significantly negative, indicating that it has reduced the resources available for local enterprises to carry out green innovation, which is in line with the results of Table 3. However, the coefficient of the spatial Durbin model was lower, at 26.331, indicating that the crowding out effect of corporate social responsibility on local enterprises’ green innovation is affected by the innovation of enterprises in surrounding cities, and the benchmark model’s estimation results take this externality into account. Based on the spatial contiguity matrix and the anti-distance matrix, the impact of corporate social responsibility on enterprise innovation in neighbouring areas is significantly negative. This implies that corporate innovation between regions has a positive correlation in the presence of regional and spatial innovation inhibition, demonstrating that corporate social responsibility reduces both local and peripheral corporate innovation. As concluded before, corporate social responsibility is a behaviour driven by the private interests of leadership, such as satisfying government regulations or stakeholder demands, which means implementation of Corporate Social Responsibility (CSR) based on external motivation can lead to opportunistic behavior and not conducive to enterprise innovation. This is because R&D expenses reduce current accounting profits, which does not benefit the remuneration of managers and provides greater returns to future successors. As a result, managers motivated by self-interest may prioritize fulfilling social responsibilities over innovation in order to obtain short-term benefits. According to empirical evidence, a higher degree of CSR fulfillment actually decreases the green innovation of the enterprise. Additionally, the imitation isomorphism theory suggests that enterprises will imitate successful companies, leading to more social responsibilities being fulfilled in the local area and a decrease in innovation. Furthermore, the “reputation effect” created by CSR can also encourage neighboring companies to follow it.

**Table 4:** The test of spatial effect.

	contiguity matrix		anti-distance matrix	
	(1)	(2)	(3)	(4)
	green	green_r	green	green_r
CSR	-26.331***	-23.048***	-27.497***	-23.715***
	(4.870)	(3.254)	(4.893)	(3.274)
Size	0.388***	0.116***	0.386***	0.116***
	(0.018)	(0.012)	(0.018)	(0.012)
Lev	-0.250***	-0.034	-0.244***	-0.034
	(0.069)	(0.046)	(0.070)	(0.047)
ROA	-0.347**	-0.160*	-0.340**	-0.159*
	(0.130)	(0.087)	(0.130)	(0.087)
Cashflow	0.043	0.012	0.046	0.015
	(0.097)	(0.065)	(0.097)	(0.065)
Growth	-0.008***	-0.002	-0.008***	-0.002
	(0.002)	(0.001)	(0.002)	(0.001)
TobinQ	0.013**	0.001	0.013**	0.001
	(0.006)	(0.004)	(0.006)	(0.004)
Dual	-0.032	-0.029**	-0.032	-0.029**
	(0.021)	(0.014)	(0.021)	(0.014)
Top1	-0.021	-0.205**	-0.032	-0.213**
	(0.106)	(0.071)	(0.106)	(0.071)
W*CSR	-48.946***	-21.182**	-126.920*	-69.558
	(14.091)	(9.450)	(65.798)	(44.015)
ρ	0.091***	0.155***	0.177	0.444***
	(0.024)	(0.021)	(0.151)	(0.127)
N	13905	13905	13905	13905

Note: The robust standard errors are used in the estimation.

* represents significance at the 10% significance level

** represents significance at the 5% significance level

*** represents significance at the 1% significance level.

### 3.5. Mechanism testing

#### 3.5.1. financial constraints

The process of innovation is long-term and persistent, and is accompanied by information asymmetry and high investment risks, with the output of innovation usually being intangible, making it difficult to evaluate the value. Banks often require companies to provide tangible collateral before granting credit, resulting in strong financing constraints for innovative activities. However, theoretical analysis has shown that reducing these financing constraints may not necessarily be beneficial for the innovative activities of businesses. The empirical results in Table 5 suggest that corporate social responsibility (CSR) has a significant negative effect with the on the financing constraints of local enterprises, while the spatial lag coefficients of CSR are significantly positive.The KZ index under the contiguity matrix has a regression coefficient of -41.459 for local effect and 89.9 for spatial effect.This implies that fulfilling social responsibility can reduce the financing constraints of local enterprises and enhance the financing constraints of surrounding enterprises. This is due to the fact that disclosing corporate social responsibility information enables stakeholders to gain a comprehensive understanding of the business’ performance, evaluate its capacity for sustainable growth, and make the enterprise more transparent. Additionally, it conveys to the market that the company is devoted to green and sustainable development, building a positive public image and decreasing the cost of obtaining external financing. When enterprise have access to more financial resources, it may lose the incentive to innovate for long-term success. Additionally, as markets become more integrated, the boundaries between regions are becoming less distinct. Companies that prioritize corporate social responsibility can draw resources from the surrounding area to their own, thus increasing the abundance of resources in the region while also limiting the financing options of other businesses and thus impacting innovation.

**Table 5:** The test of financing constraint mechanism.

	contiguity matrix		anti-distance matrix	
	(1)	(2)	(3)	(4)
	KZ	WW	KZ	WW
CSR	-41.459***	-2.382***	-42.036***	-2.380***
	(5.736)	(0.175)	(5.733)	(0.175)
Size	-0.262***	-0.048***	-0.271***	-0.048***
	(0.022)	(0.001)	(0.022)	(0.001)
Lev	5.868***	0.031***	5.820***	0.031***
	(0.089)	(0.003)	(0.089)	(0.003)
ROA	-3.183***	-0.109***	-3.197***	-0.109***
	(0.184)	(0.006)	(0.184)	(0.006)
Cashflow	-11.856***	-0.090***	-11.876***	-0.090***
	(0.128)	(0.004)	(0.128)	(0.004)
Growth	-0.004*	-0.035***	-0.004	-0.035***
	(0.002)	(0.000)	(0.002)	(0.000)
TobinQ	0.342***	0.001**	0.341***	0.001**
	(0.010)	(0.000)	(0.010)	(0.000)
Dual	-0.072**	-0.002**	-0.069**	-0.002**
	(0.027)	(0.001)	(0.027)	(0.001)
Top1	0.048	-0.015***	0.110	-0.015***
	(0.130)	(0.004)	(0.130)	(0.004)
W*CSR	89.900***	2.038***	205.997**	7.266***
	(15.024)	(0.460)	(69.724)	(2.017)
ρ	0.117***	0.031**	0.668***	0.164***
	(0.015)	(0.012)	(0.063)	(0.047)
N	9720	9720	9720	9720

Note: The robust standard errors are used in the estimation.

* represents significance at the 10% significance level

** represents significance at the 5% significance level

*** represents significance at the 1% significance level.

#### 3.5.2. External governance

The Principal-Agent Theory suggests that disclosing information about corporate social responsibility can reduce the cost of external stakeholders to gather data, allowing them to better monitor management. It also encourages management to prioritize green innovation and prevent a lack of resources due to moral hazard. Additionally, it can minimize the negative effects of conflicting interests between management and shareholders on green innovation, thus providing a form of governance. By taking on their social responsibilities and improving their social reputation capital, enterprises can gain the favor of local investors and draw the investment attention and willingness of surrounding investors to congregate locally. As external stakeholders’ attention is a scarce resource in market competition, this reduces the likelihood of surrounding enterprises receiving attention and makes it hard for external stakeholders to exert their governance and supervision.

The empirical results in Table 6 show that the coefficient of CSR on external governance is significantly positive, while the coefficient of CSR spatial lag is significantly negative. In regards to the INST under the adjacency weight matrix, the regression coefficient of local effect is 2.287 and the regression coefficient of spatial effect is -13.761.This suggests that when companies fulfill their social responsibility, it can significantly enhance the external governance effect of local enterprises and reduce the external governance effect of surrounding enterprises.

**Table 6:** The test of external governance mechanism.

	contiguity matrix		anti-distance matrix	
	(1)	(2)	(1)	(2)
	INST	Indep	INST	Indep
CSR	2.287**	0.612**	1.869**	0.519*
	(0.800)	(0.294)	(0.824)	(0.295)
Size	0.039***	-0.003**	0.039***	-0.003**
	(0.003)	(0.001)	(0.003)	(0.001)
Lev	-0.002	-0.006	-0.007	-0.007
	(0.011)	(0.005)	(0.012)	(0.005)
ROA	0.115***	-0.006	0.116***	-0.005
	(0.021)	(0.009)	(0.022)	(0.009)
Cashflow	-0.054***	-0.002	-0.052**	-0.002
	(0.016)	(0.007)	(0.016)	(0.007)
Growth	-0.001**	-0.000	-0.000	-0.000
	(0.000)	(0.000)	(0.000)	(0.000)
TobinQ	0.024***	0.000	0.024***	0.000
	(0.001)	(0.001)	(0.001)	(0.001)
Dual	0.006*	0.009***	0.006*	0.009***
	(0.003)	(0.001)	(0.003)	(0.001)
Top1	0.166***	0.013**	0.169***	0.013**
	(0.017)	(0.007)	(0.018)	(0.007)
W*CSR	-13.761***	-1.524**	-19.451*	0.930
	(2.310)	(0.728)	(9.981)	(3.460)
ρ	0.191***	0.011	0.234*	-0.151
	(0.022)	(0.022)	(0.127)	(0.186)
N	13905	9711	13392	9711

Note: The robust standard errors are used in the estimation.

* represents significance at the 10% significance level

** represents significance at the 5% significance level

*** represents significance at the 1% significance level.

#### 3.5.3. Political connection

Research has demonstrated that the implementation of corporate social responsibility can impede corporate innovation, and this is often due to external policy effects, which can lead to a shift in the level of political involvement of businesses. As a significant part of corporate political resources, political ties may distort the allocation of resources by the government, thus hindering technological innovation by companies. This article will explore whether the execution of corporate social responsibility will affect the innovation activities of businesses by influencing political connections. Companies with more plentiful political resources are more likely to engage in rent-seeking activities to enhance their performance, which reduces the incentive for the management of politically connected companies to improve their performance through innovative activities. Executives with political connections can use their influence to shift regulations that are not beneficial to their business to other areas, thus impeding the green innovation of other businesses. To build a positive corporate image that is in line with the government’s public goals, companies should fulfill their tax obligations, improve the working environment and welfare of their employees, promote environmental protection and philanthropy, and adhere to quality standards. These actions will help to foster social stability and the realization of social objectives, and will also increase the political relevance of the company. The findings of this study demonstrate that corporate social responsibility (CSR) can have a significant impact on the political connections of local and surrounding enterprises. The results of Table 7 showed that the coefficient of CSR was significantly positive at the 5% level, and the spatial lag coefficient of CSR was also significantly positive. For example, the regression coefficient of local effect for the PC under the adjacency weight matrix is 4.497, and the regression coefficient of spatial effect is 15.066.This indicates that fulfilling CSR can significantly promote the political connections of both local and surrounding enterprises. Additionally, CSR was found to significantly improve the political affiliation level of surrounding enterprises, but not of local enterprises. In conclusion, fulfilling CSR can significantly increase the political connections between local and surrounding enterprises, as well as enhance the political connection level of the latter.

**Table 7:** The test of political connection.

	contiguity matrix		anti-distance matrix	
	(1)	(2)	(3)	(4)
	PC	PClevel	PC	PClevel
CSR	4.497**	10.193	4.747**	11.344
	(2.249)	(7.374)	(2.258)	(7.407)
Size	0.018**	0.104***	0.018**	0.105***
	(0.008)	(0.028)	(0.008)	(0.028)
Lev	0.071**	0.250**	0.072**	0.255**
	(0.032)	(0.105)	(0.032)	(0.106)
ROA	-0.041	-0.025	-0.041	-0.026
	(0.060)	(0.196)	(0.060)	(0.196)
Cashflow	-0.003	0.055	-0.003	0.052
	(0.045)	(0.147)	(0.045)	(0.147)
Growth	0.003**	0.005	0.003**	0.005
	(0.001)	(0.003)	(0.001)	(0.003)
TobinQ	-0.007**	-0.018*	-0.007**	-0.018*
	(0.003)	(0.009)	(0.003)	(0.009)
Dual	-0.089***	-0.250***	-0.089***	-0.252***
	(0.010)	(0.031)	(0.010)	(0.031)
Top1	0.030	0.331**	0.030	0.331**
	(0.049)	(0.160)	(0.049)	(0.160)
W*CSR	15.066**	77.960***	30.588	196.699*
	(6.505)	(21.305)	(30.651)	(101.273)
ρ	0.056**	0.060**	0.183	0.103
	(0.024)	(0.024)	(0.160)	(0.164)
N	13905	13905	13905	13905

Note: The robust standard errors are used in the estimation.

* represents significance at the 10% significance level

** represents significance at the 5% significance level

*** represents significance at the 1% significance level.

## 5. Conclusion and enlightenment

Environmental pollution is a pressing global issue that requires collaborative solutions. This paper uses Panel data from 293 Prefecture-level cities in China from 2010 to 2020 to analyze the spatial spillover effect of corporate social responsibility on corporate green innovation. The results suggest that corporate social responsibility, driven by external “tool” motivation, has a positive effect on green innovation and a spillover effect on surrounding areas. This may be due to its ability to alleviate financing constraints, improve external governance effects, and gain political connections. The findings of this study have important implications for policy makers.

1. Regulatory authorities must take steps to avoid credit rent-seeking distorting the distribution of credit resources, which can ease financing constraints but hamper enterprise innovation. On the one hand, financial regulatory agencies should strive to further enhance the financial market system, allowing the market to take the lead in resource allocation, so as to reduce the incentive for micro enterprise credit rent-seeking, address the financing issues of innovative businesses, and ultimately drive enterprise innovation. On the other hand, it is of great important to create a sustainable system to manage financial rent-seeking, eliminate the relationships between banks and enterprises based on money, reinforce external audits and monitoring of banks, and reduce the rent-seeking activities of bank personnel.
2. Institutional investors should cultivate a long-term investment and value investing mindset, seek out industries and businesses with potential for growth, make informed decisions, and benefit from the success of the companies they invest in. Furthermore, they should be aware of their responsibility to the capital market, take part in the governance of listed companies, comply with national regulations, and push companies to be socially responsible and prioritize innovation.
3. The government should strive to create strong and trustworthy political and commercial ties, and should not seek personal gain or be involved in any form of bribery. It should take into account the challenges faced by businesses and work to resolve the issues that impede innovation. To foster innovation in businesses, we should take measures such as reducing taxes and regulations, setting up subsidy systems, and allowing the market to play a greater role in resource allocation. This will reduce the government’s direct involvement in enterprise decision-making and encourage the efficient circulation and allocation of innovation resources. Additionally, the government should ensure a fair competitive environment and provide public services to create a favorable atmosphere for innovation.

This article’s research has a couple of limitations: Firstly, green innovation should be considered on a wider scale, but the article only looks at green technology innovation through the lens of patent numbers, not taking into account green institutional and cultural innovation. In order to have a more complete understanding of green innovation, the concept needs to be further explored to comprehend the different impacts from corporate social responsibility. Secondly, the article only provides a theoretical explanation for why corporate social responsibility can both promote and inhibit green innovation, with the net effect being negative. Further empirical research should be conducted to determine if the relationship between corporate social responsibility and green innovation is more complex than a simple linear one.

## Supporting information

S1 File (DTA)
